# Clinical Characteristics and Outcomes of Aortic Arch Emergencies: Takayasu Disease, Fibromuscular Dysplasia, and Aortic Arch Pathologies: A Retrospective Study and Review of the Literature

**DOI:** 10.3390/biomedicines11082207

**Published:** 2023-08-06

**Authors:** Magdalena Wawak, Łukasz Tekieli, Rafał Badacz, Piotr Pieniążek, Damian Maciejewski, Mariusz Trystuła, Tadeusz Przewłocki, Anna Kabłak-Ziembicka

**Affiliations:** 1 Department of Interventional Cardiology, The John Paul II Hospital, Prądnicka 80, 31-202 Kraków, Poland; 2 Department of Interventional Cardiology, Institute of Cardiology, Jagiellonian University Medical College, św. Anny 12, 31-007 Kraków, Poland; 3 Department of Cardiac and Vascular Diseases, Institute of Cardiology, Jagiellonian University Medical College, św. Anny 12, 31-007 Kraków, Poland; 4 Department of Vascular and Endovascular Surgery, The John Paul II Hospital, Prądnicka 80, 31-202 Kraków, Poland; mariusz.trystula@szpitaljp2.krakow.pl; 5 Noninvasive Cardiovascular Laboratory, The John Paul II Hospital, Prądnicka 80, 31-202 Kraków, Poland

**Keywords:** aortic arch pathology, aortic dissection, arteritis, connective tissue disorders, fibromuscular dysplasia, percutaneous intervention, rare cardiovascular disease, stenosis of aortic arch, Takayasu disease

## Abstract

Non-atherosclerotic aortic arch pathologies (NA-AAPs) and anatomical variants are characterized as rare cardiovascular diseases with a low incidence rate, below 1 case per 2000 population, but enormous heterogeneity in terms of anatomical variants, i.e., Takayasu disease (TAK) and fibromuscular dysplasia (FMD). In specific clinical scenarios, NA-AAPs constitute life-threatening disorders. Methods: In this study, 82 (1.07%) consecutive patients with NA-AAPs (including 38 TAKs, 26 FMDs, and 18 other AAPs) out of 7645 patients who underwent endovascular treatment (EVT) for the aortic arch and its side-branch diseases at a single institution between 2002 and 2022 were retrospectively reviewed. The recorded demographic, biochemical, diagnostic, operative, and postoperative factors were reviewed, and the functional outcomes were determined during follow-up. A systematic review of the literature was also performed. Results: The study group comprised 65 (79.3%) female and 17 (21.7%) male subjects with a mean age of 46.1 ± 14.9 years. Overall, 62 (75.6%) patients were diagnosed with either cerebral ischemia symptoms or aortic arch dissection on admission. The EVT was feasible in 59 (72%) patients, whereas 23 (28%) patients were referred for medical treatment. In EVT patients, severe periprocedural complications occurred in two (3.39%) patients, including one periprocedural death and one cerebral hyperperfusion syndrome. During a median follow-up period of 64 months, cardiovascular events occurred in 24 (29.6%) patients (5 deaths, 13 ISs, and 6 myocardial infarctions). Repeated EVT for the index lesion was performed in 21/59 (35.6%) patients, including 19/33 (57.6%) in TAK and 2/13 (15.4%) in FMD. In the AAP group, one patient required additional stent-graft implantation for progressing dissection to the iliac arteries at 12 months. A baseline white blood count (odds ratio [HR]: 1.25, 95% confidence interval [CI]: 1.11–1.39; *p* < 0.001) was the only independent prognostic factor for recurrent stenosis, while a baseline hemoglobin level (HR: 0.73, 95%CI: 0.59–0.89; *p* = 0.002) and coronary involvement (HR: 4.11, 95%CI: 1.74–9.71; *p* = 0.001) were independently associated with a risk of major cardiac and cerebral events according to the multivariate Cox proportional hazards regression analysis. Conclusions: This study showed that AAPs should not be neglected in clinical settings, as it can be a life-threatening condition requiring a multidisciplinary approach. The knowledge of prognostic risk factors for adverse outcomes may improve surveillance in this group of patients.

## 1. Introduction

Non-atherosclerotic aortic arch pathologies (NA-AAPs) and its anatomical variants are usually a set of symptoms that escapes the attention of clinicians [1,2,3]. However, in specific clinical scenarios, pathologies of the aortic arch and its side-branches constitute a life-threatening disorder. NA-AAPs are characterized as rare cardiovascular diseases, with a low incidence rate below 1 case per 2000 population, but a substantial etiological heterogeneity that includes anatomical variants and aortopathies, Takayasu disease (TAK), dissection, and fibromuscular dysplasia (FMD) [4,5].

TAK is defined as granulomatous inflammation of the aorta and its major branches, causing artery narrowing or aneurysms [6]. TAK’s prevalence is estimated at 1–4.7 person per 1 million population in Europe [7,8]. The disease’s location determines a variety of clinical symptoms which present on examination of a patient, including the most prevalent ones, like pulselessness over the radial artery, claudication, arterial hypertension, or complicated pregnancy [8,9,10]. Clinical manifestations such as fevers, weight loss, pain in the affected arteries, elevated inflammatory markers, anemia, and thrombocytopenia accompany the active phase of the disease. Sadly, the majority of TAK diagnoses are made after a sudden ischemic vascular event, including ischemic stroke (IS), myocardial infarction (MI), or other cardiovascular events in surprisingly young patients [8,9,11]. 

FMD is a non-atherosclerotic, non-inflammatory disease that affects middle-size arteries, including the carotid, vertebral, renal, mesenteric, pulmonary, and coronary arteries, but similarly to TAK, the FMD is typically recognized when a cardiovascular event occurs [12,13,14]. Unilateral head/neck pain or focal neurologic findings (e.g., partial Horner’s syndrome with ipsilateral ptosis and miosis) is suggestive of a carotid artery dissection [13]. However, patients can present clinical symptoms for years before a cardiovascular event occurs [12,13,14]. FMD in the carotid and vertebral locations is associated with migraines, blurred vision, pulsatile tinnitus, and vertigo, while arterial hypertension and angina are associated with renal and coronary involvements [15,16]. 

Other NA-AAPs comprise a heterogeneous entity resulting in abrupt dissection of the wall of the aortic arch, as well as associated acute cerebrovascular and/or cardiovascular symptoms [3,4,5,17,18]. Again, non-specific chronic clinical symptoms are often present for years [17,18]. The NA-AAPs include anatomical variants of the aortic arch, like a right-sided aortic arch with variants in the sequence and course of aortic side-branches (prevalence 1/2000), a bovine arch, an aberrant right subclavian artery (arteria lusoria; prevalence 0.2–2.0% in general population), coarctation of the aorta (CoA), and aortopathies such as vascular type of Ehlers-Danlos syndrome (prevalence 5.0/100,000) [18,19,20,21]. Dysphagia, choking, swelling difficulties, cough, dyspnea, and respiratory tract infections are associated with a retrograde course of the aberrant subclavian artery behind the trachea that compresses the esophagus [18,19,20,22]. Vascular Ehlers–Danlos usually co-exists with the other connective tissue disorders, like ocular, joint, and muscle diseases, which facilitates diagnosis [5,20].

The progress made in the development of the diagnostic work-ups, including color Doppler ultrasonography (CDUS), computed tomography angiography (CTA), magnetic resonance imaging (MRI), positron emission tomography (PET), and therapeutic work-up facilitates the recognition of NA-AAPs [4,14,22,23,24]. However, physicians’ awareness of such pathologies and of clinical symptoms (often present since childhood), which might lead to cerebro-cardiovascular events, is essential for patients’ referral to the aforementioned diagnostic work-ups. 

Eventually, endovascular treatment (EVT) and surgical approaches might be necessary in the light of stenosis and occlusions that cannot be resolved by conservative approaches [25,26,27,28]. Long-term prognosis depends on the applied EVT or surgery, accompanied by the specific pharmacological treatment that allows for the optimization of patient care. These treatments provide hope for favorable outcomes in individual patients [25,26,27,28].

Therefore, in this study, we retrospectively reviewed cases of consecutive patients with NA-AAP. The study aimed to assess the long-term outcomes in patients with a diagnosed TAK, FMD, or other NA-AAP who were referred to our institution for the EVT procedure. A systematic review of the literature was also performed.

## 2. Materials and Methods

### 2.1. Study Population

**Figure 1:**
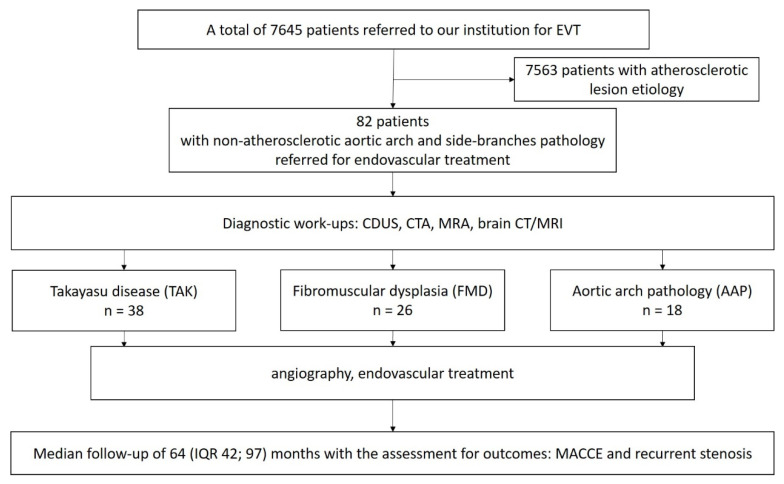
Study flow chart.

A total of eighty-two (1.07%) consecutive patients with NA-AAPs out of 7645 patients referred to EVT for diseases of the aortic arch and its side-branches between 2002 and 2022 at a tertiary hospital (the John Paul II Hospital, Krakow, Poland) were retrospectively reviewed. The study’s flow chart is presented in Figure 1. Patients were divided into three categories: TAK disease, FMD, and other AAPs.

**Figure 2:**
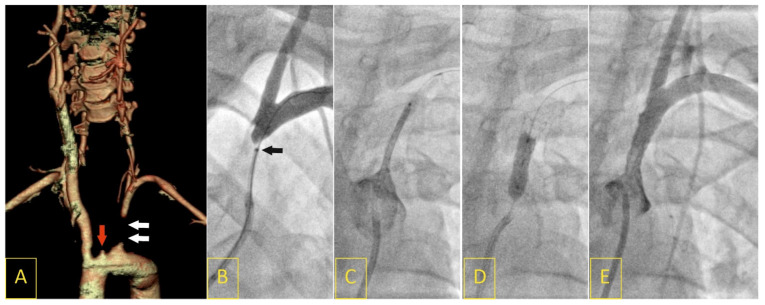
A 17-year-old female presented with “stuttering” symptoms of left-hemisphere ischemia (8 points on the National Institute of Health Stroke Scale upon admission). Computed tomography angiography showed a proximal left subclavian artery (LSA) occlusion (white arrows) with coexisting ostial left internal carotid artery occlusion (red arrow, (**A**)). The LSA occlusion segment was crossed with a V-18 Control Wire with a support of a NaviCross 4F catheter (tip of the support catheter marked with a black arrow (**B**)). Following predilatation, a drug-eluting balloon-mounted stent (Nefro 8 × 30) was implanted and postdilated (**C**,**D**). Angiography showed an optimal result of the procedure, with normal flow of the vertebral artery (**E**).

The TAK group comprised 38 consecutive subjects (35 female, 3 male) with non-atherosclerotic large artery lesions with aortic arch involvements, with a mean age of 43.2 ± 13.5 years (range of 17–56), who were referred to EVT for stenosis of the aortic arch side-branches. The diagnosis of TAK was made using the American College of Rheumatology criteria [29]. Eleven (28.9%) patients were referred as urgent cases. A recent cerebral ischemia event (past 6 months) was recorded in 23 (60.5%) patients. Active arteritis was reported in 27 (71.1%) patients at the time of EVT. Figure 2 demonstrates a typical clinical scenario of urgent hospitalization of a patient with TAK.

**Figure 3:**
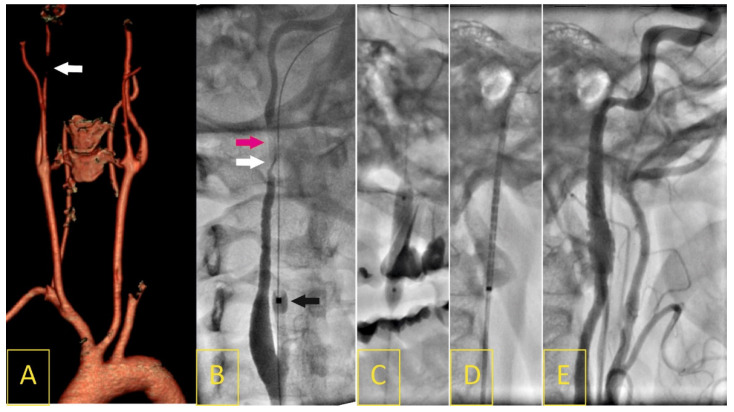
A 45-year-old woman who had been unsuccessfully treated for several years for headaches and “swishing” noises in her right ear presented with a 12-day history of right-hemisphere ischemic stroke (4 points in the National Institute of Health Stroke Scale). A Doppler ultrasound image on admission revealed long, irregular, string-sign right internal carotid artery (RICA) stenosis in its intracranial segment, with a peak systolic velocity of 5.28 m/s and end-diastolic velocity of 2.53 m/s, corresponding to lumen stenosis of more than 90%. Computed tomography angiography confirmed a 5 cm long 95% RICA stenosis with “string-of-beads” morphology typical for FMD (**A**). Catheter angiography showed RICA string-sing stenosis (white arrow) with local dissection (pink arrow, (**B**)); black arrow indicates distal balloon of proximal neuroprotection system (9F Mo.Ma). Under flow reversal, the stenosis/dissection segment was crossed with a 0.014” wire, then predilated with a 4 × 20 mm semi-compliant balloon (**C**). A self-expanding 8 × 30 mm stent (Precise) was deployed (**D**). Angiography (**E**) demonstrated effective reconstruction of the RICA lumen with significant improvement to the distal flow (TICI-3).

The FMD group comprised 26 consecutive patients (17 females, 9 males) of a mean age of 50.1 ± 15.3 (range of 19–72 years) with stenosis of the middle-distal portion of the internal carotid artery involving intracranial segments, including 14 (53.8%) patients with cervical artery dissection on admission. A recent cerebral ischemia was documented in 20 (76.9%) patients (Figure 3).

**Figure 4:**
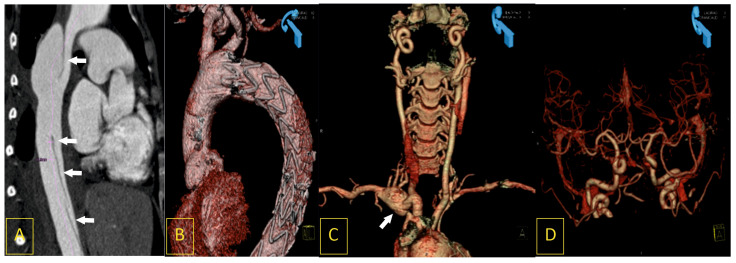
A 28-year-old woman, ten days after the uneventful delivery of a healthy daughter, was admitted to the cardiac emergency unit after a short episode of syncope with severe chest pain and symptoms of deteriorating cardiac shock. Computed tomography angiography (CTA) revealed acute type III dissection of the thoracic aorta (**A**). It was successfully treated with immediate implantation of endovascular stent-graft prosthesis (**B**). The same CTA revealed other vascular pathologies that may be found in Ehlers–Danlos syndrome: an aneurysm of the right subclavian artery ((**C**), white arrow) and arterial loops caused by elongation of the intracranial segments of the internal carotid arteries (**D**).

The AAP group comprised 18 patients (13 females, 5 males) of a mean age of 46.4 ± 25.7 (range of 19–74 years), including 16 (88.9%) patients who presented on admission with either cerebral ischemia, cardiogenic shock, or both resulting from a dissecting aortic arch or the major aortic arch side-branches. The AAP group consisted of six patients with right-sided aortic arches and arteria lusoria, six patients with bovine arches, one patient with a bicarotid trunk, one patient with hypoplasia of the aortic arch, three patients with variants of the aortic arch and CoA, and one patient with Ehlers–Danlos syndrome. In this group, 4 female patients developed symptoms of cardio-cerebral events in the peripartum or postpartum period, between the 1st and 14th day after delivery of a child (Figure 4). 

The final diagnosis of artery status was based on CDUS, CTA, or angio-MRI followed by conventional angiography, which is the gold standard, and EVT when feasible. The etiology was assessed from the available past medical history, or from further consultations with the rheumatology and/or immunology departments.

Patients with a presumed atherosclerotic etiology, ascending aorta aneurysm, bicuspid aortic valve, congenital heart diseases (like the Tetralogy of Fallot), genetic syndromes (like Marfan, Down, and Noonan syndrome), or trauma-related aortic dissection and/or rupture were not included in this analysis. 

All patients were assessed for the prevalence of cardiovascular risk factors. Definitions of the above were adopted from the scientific statements of the European Society of Cardiology (http://www.escardio.org (accessed on 15 June 2023)). Furthermore, the results of lipid levels, serum creatinine, white blood count, hemoglobin level, and inflammatory and thrombotic process activity (hs-CRP, white blood count, D-dimers) were analyzed. 

The study was supported by a national grant, and the protocol was reviewed and approved by the local ethical committee (2PO5B09330). Each patient provided written consent to participate in the study prior to enrollment. The study was conducted in accordance with the Declaration of Helsinki of 1975 and was approved by the local Ethics Committee.

### 2.2. Definitions and Confirmation of the Diagnosis

The other arterial locations specific for the disease were verified with CDUS, CTA, or angio-MRI in order to assure a correct diagnosis.

FMD was recognized when beaded multifocal (string-of-beads) and focal/tubular lesions other than plaques in medium-sized arteries were displayed [30]. Arterial dissection and/or an aneurysm localized in a medium-sized artery was considered FMD if a patient had a focal or multifocal lesion(s) in another/other vascular bed(s) (Figure 2). The definition was adopted from the First International Consensus on the diagnosis and management of FMD [13]. 

For TAK, the individual vessels involved in angiography, as well as the overall angiographic subtype of TAK, were recorded in accordance with Hata’s classification [31]. In this study, patients with type I, II, or V were enrolled, which was consistent with the aortic arch involvement (Figure 3) [31]. The TAK diagnosis had to be confirmed by a consultant in immunology or rheumatology, and the diagnosis had to be based on an imaging modality like CTA, angio-MRI, or PET, as well as on the results of additional diagnostic disease-specific laboratory tests [32].

AAP was established based on radiological images of CTA or angio-MRI, and they were defined as a bovine arch, vascular ring, right aortic arch, presence of arteria lusoria, Kommorell aneurysm, co-existing CoA, hypoplasia of the aortic arch, or vasculopathy (Figure 4). The aortic arch types were classified according to the Adachi–Williams Classification [21]. A right aortic arch was recognized when the aortic arch traversed over the right bronchus instead of the left bronchus. There are scenarios in which a right aortic arch is clinically relevant [19]. 

Neurological examination was provided for all subjects by a consultant neurologist on admission. At the same time, the data on IS or transient ischemic attack (TIA) were obtained from a stroke unit and sourced from the available medical documentation. Data were also obtained from brain imaging, either with CT scans or MRI, to assess the degree of cerebral infarction, if any, and to exclude other disorders that might have been causing the symptoms (e.g., subdural hematoma, intracranial hemorrhage, tumor, etc.). 

### 2.3. Diagnostic Work-Ups

CDUS scanning was performed with a high-resolution Toshiba Aplio PowerVision ultrasound machine (Toshiba Medical Systems Co., Ltd., Tokyo, Japan) equipped with a 4–11 MHz linear-array transducer and a 3.5–5.0 MHz convex transducer. The stenosis grade of the supraaortic arteries was based on the increase in the peak systolic velocity and the end-diastolic velocity, and the detection of steal syndrome was based on the flow direction in the vertebral arteries [33,34]. 

CTA image acquisition was carried out with a 64-multi-detector-row CT system (Somatom 64, Siemens, Erlangen, Germany) using a routine imaging protocol. Biplanar and 3-dimensional reconstructions of the vessels were computed to characterize the anatomy of the aorta and its main branches [35].

MRI angiography was performed with a 1.5 T scanner (Magnetom Sonata Maestro Class, Erlangen, Germany) using a dedicated 8-element head coil [36].

A selective digital angiography of the index artery or arteries was performed using a Coroscop or Axiom Artis Zee angiograph (Siemens, Germany) with multiple angulated projections [33,35,36].

### 2.4. Patients Management and Procedures

Patients were referred to EVT with medical treatment on the basis of a multidisciplinary working team including a consultant neurologist, vascular surgeon, endovascular specialist, cardiologist, and radiologist. The decision was reached by taking into account the clinical presentation, accompanying comorbidities (e.g., renal dysfunction, respiratory tract disease, access site), anatomic assessment (limited surgical access), data from imaging workups, and method feasibility and safety. In patients referred to EVT, a decision on the method of revascularization, e.g., with or without stent implantation, the use of a neuroprotection system (NPS), anticoagulation, etc., was left to the operator’s discretion. 

For stenosed arteries, EVT was performed using the femoral or radial approach. Patients were preloaded with anti-platelets including aspirin, clopidogrel, and heparin periprocedurally. A loading dose of aspirin of 300 mg followed by 75 mg o.d., and a loading dose of clopidogrel of 600 mg followed by 75 mg o.d., was administered for 6 months. During the procedure, unfractionated heparin was administered according to each patient’s weight and activated clotting time. Other medications, such as analgesics, atropine, and beta-blockers, were administered as indicated. 

During the revascularization procedure, the target vessel was routinely stented in TAK patients. Predilatation and stent choice depended on the clinical circumstances and the operator’s decision. In the case of dissecting FMD, a decision regarding whether to perform angioplasty with a provisional stenting or not depended on the visualization of a false-true lumen of the index lesion and its technical feasibility. Uncomplicated FMDs were primarily referred for balloon angioplasty and stented in case of a dissection complicating the procedure. 

In the AAPs group, for a dissecting aortic arch (n = 7), a stent-graft was implanted, whereas for a dissecting or stenosed carotid, innominate, or subclavian artery at origin, stent implantation was the primary choice. It was the operator’s decision as to whether or not to use the cerebral neuroprotection system (NPS). The detailed EVT technique was left to the operator’s discretion, provided that the “tailored-CAS” algorithm was applied, allowing for an optimal choice of NPS and stent type depending on the lesion, anatomical conditions, intracranial collateral flow, and the neurological status of the patient [37]. The detailed EVT approach has been described previously [37,38]. 

All patients obtained medical treatment according to guidelines of the European Society of Cardiology, the Society of Neurology, and the Society of Rheumatology when applicable [39,40,41]. 

### 2.5. Patient Follow-Up and Cardiovascular Outcomes

The incidences of restenosis (RS), cardiovascular death (CVD), myocardial infarction (MI), IS/TIA, and new lesions were recorded during a median follow-up period of 64 months (interquartile range of 42; 97 months). A follow-up was performed for all patients.

RS was defined as a recurrence of at least a 50% reduction in the arterial lumen diameter within the stent or adjacent 5 mm, or in the segment treated with balloon angioplasty. When a new symptomatic stenosis of over 50% was detected outside of the index-segment, in any of the coronary, carotid, subclavian, vertebral, renal, and lower extremity arteries, a diagnosis of a new stenotic lesion was established.

The incidences of CVD, MI, and IS, as well as a composite endpoint, i.e., major adverse cardiac and cerebral events (MACCEs), were recorded retrospectively in 81 patients. The minimum follow-up period was eleven months due to CVD. MI was diagnosed according to the criteria of the European Society of Cardiology. The diagnosis of a cerebral event was to be given by a neurologist in order to ensure reliability. CVD was defined as a fatal IS, fatal MI, or other CVD (i.e., any sudden or unexpected death unless proven to be non-cardiovascular on autopsy).

The final patient status was confirmed from the National Health Institute registry.

### 2.6. Statistical Analysis

Continuous variables are presented as the mean ± one standard deviation, and categorical variables are expressed as frequencies and percentages. The means of analyzed parameters across groups were tested with an analysis of variance (ANOVA) test, and the frequencies were compared using the chi square test for independence. The normal distribution of the studied variables was determined by means of the Shapiro–Wilk test. The differences between mean values were verified using the Student *t*-test, since the distribution of variables was found to be normal.

The potential independent prognostic markers of MACCE and RS during the follow-up period were established from the 30 variables with univariate Cox proportional hazard analysis (including demographic, biochemical, diagnostic, operative, and postoperative factors). If there was a trend toward a difference (*p* < 0.1), they were entered into a multivariate Cox proportional hazard analysis model. The results of the multivariate logistic regression analysis were expressed as hazard ratio (HR) and 95% confidence interval (95% CI). Kaplan–Meier free survival curves from RS and MACCE were constructed to compare the survival between the studied groups (a log-rank test). 

Statistical analyses were performed using Statistica 12.0 software. Statistical significance was assumed at *p* < 0.05.

## 3. Results

### 3.1. Patient Characteristics

A total of 82 (1.07%) consecutive patients with NA-AAPs (including 38 TAKs, 26 FMDs, and 18 other AAPs) out of 7645 patients referred to EVT for diseases of the aortic arch and its side-branches at our institution between 2002 and 2022 were included in this study. The majority of patients with NA-AAPs were admitted on an urgent basis, presenting with either acute cerebral ischemia, aortic arch or cervical artery dissection, or cardiogenic shock. The detailed characteristics of the patients are presented in Table 1.

In 30 (78.9%) out of 38 TAK patients, chronic symptoms had been reported previously, such as upper and/or lower limb claudication (n = 18), chronic inflammation (n = 29), fevers (n = 25), headaches or migraines (n = 11), ocular disturbances (n = 6), resistant hypertension (n = 10), abdominal pain (n = 2), pulse deficiency (n = 15), and syncope (n = 3). In the FMD group, 11 (42.3%) out of 26 patients complained about chronic symptoms, including migraines (n = 11), blurred vision (n = 5), and resistant hypertension (n = 7). In the AAP group, chronic symptoms were reported by 11 (61.1%) out of 18 patients, the most often reported symptoms being dysphagia and swelling discomfort (n = 3), dyspnea (n = 2), resistant hypertension (n = 4), blurred vision and migraines (n = 3), and claudication (n = 5).

Across all groups, a predominance of female patients was observed. Diabetes (28.9%), hyperlipidemia (60.5%), and past or current cigarette smoking (36.8%) were more frequent in the TAK group as compared to the FMD group (11.5%, 46.1%, and 7.6%, respectively) and the AAP group (16.6%, 50%, and 22.2%, respectively). The distributions of age, hypertension, chronic kidney disease, and body mass index were similar across all study groups. TAK and AAP patients presented with elevated levels of hs-CRP and D-dimers on admission. Other immune-mediated diseases, such as Crohn’s disease, colitis ulcerosa, and Hashimoto disease, were diagnosed in 11 (28.9%) patients with TAK and 1 (3.8%) patient with FMD. 

Coronary or cardiac involvements were observed more often in the TAK group compared to the FMD and AAP groups. Moreover, all patients with TAK were on anti-inflammatory therapy, mainly with steroids (n = 38), whereas cyclophosphamide, methotrexate, azathioprine, archine, and/or tocilizumab alone or in combination were being taken by 17 patients.

In the FMD group, a significant narrowing of the coronary and abdominal arteries was observed in 11.5% and 61.5% of the patients, respectively. In the AAP group, cardiac involvement included significant coronary stenosis in three (16.6%) and a significant mitral/aortic valve disease in one (5.5%). 

### 3.2. Endovascular Procedure and Periprocedural Outcomes

The EVT was feasible in 59 (72%) patients, whereas 23 (28%) patients were referred for medical treatment. The reason for which patients were excluded from EVT was acute cervical artery dissection causing lumen occlusion (10 out of 14 patients presented with a dissecting cervical artery on admission). The failed recognition of true from false lumen limited the number of patients referred to EVT, in particular in the FMD group. Thrombolysis was administrated in three patients with dissecting FMD. Open surgery was not recommended in any of patients due to a higher perioperative risk of major complications compared to EVT. 

A total of 70 EVT procedures were performed in 59 patients, including 41 in TAK, 15 in FMD, and 14 in other AAP. There were 37 carotid artery, 26 subclavian or innominate artery, and 7 stent-graft procedures performed. In eleven patients, multiple procedures for more than one artery was performed. Stent implantation was performed in all patients except for one with FMD. In the AAP group, a stent graft was implanted in seven patients for the aortic arch and descending aorta dissection. In AAP group, four patients also had stent implantation into the aortic arch side-branches.

In EVT patients, severe periprocedural complications occurred in two (3.39%) patients, including one periprocedural death due to multi-organ failure following stent-graft implantation and one cerebral hyperperfusion syndrome following re-canalization of the subclavian artery in a patient with TAK. 

### 3.3. Recurrent Stenosis and Cardiovascular Outcomes

During a median follow-up period of 64 months, MACCE occurred in 24 (29.6%) patients, including 5 CVD, 13 non-fatal ISs, and 6 non-fatal MIs (Table 1). 

RS occurred in 21/59 (35.6%) patients, which resulted in repeated EVT for the index lesion, including 19/33 (57.6%) in TAK and 2/13 (15.4%) in FMD (*p* = 0.01). In 40% of TAK patients with a primary restenosis, RS was recurrent. 

There was no recurrence in the AAP group, although one patient required an additional stent-graft implantation for progressing dissection to iliac arteries at 12 months. Two patients developed new lesions requiring new intervention (one for a subclavian aneurysm and one for the right common carotid aneurysmal sac). 

**Figure 5:**
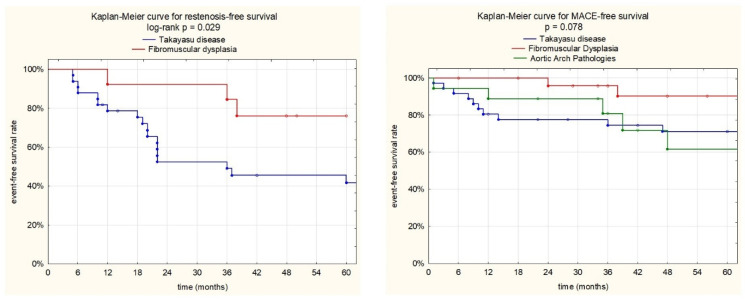
Kaplan–Meier event-free survival curves. A: Restenosis-free survival for the TAK and FMD groups. At 1, 3, and 5 years, patient free-survival rates from restenosis were 78.3%, 52.2%, and 45.4% for the TAK group and 100%, 84.3%, and 75.8% for the FMD group (log rank *p* = 0.029). B: MACCE-free survival for the TAK, FMD, and AAP groups. At 1, 3, and 5 years, patient free-survival rates from MACCE were 83.3%, 77.5%, and 71% for TAK group; 100%, 95.5%, and 89.9% for the FMD group; and 88.2%, 81.2%, and 60.9% for AAPs (log rank *p* = 0.078).

Kaplan–Meier restenosis-free-survival and MACCE-free-survival curves for the studied groups are presented in Figure 5.

### 3.4. Predictors of Restenosis and MACCE

Among the analyzed demographic, biochemical, diagnostic, operative, and postoperative variables, the patients with MACCE and restenosis differed from those with uneventful observations according to a univariate Cox proportional hazards analysis (Table 2 and Table 3).

The univariate Cox proportional hazard analysis indicated several parameters that may have had an impact on the increased risk of MACCE, including age (HR, 1.03; 95% CI, 1.01–1.07; *p* = 0.019), baseline hemoglobin level (HR, 0.78; 95% CI, 0.64–0.96; *p* = 0.019), baseline white blood count (HR, 1.10; 95% CI, 1.01–1.21; *p* = 0.045), and coronary artery involvement (HR, 2.31; 95% CI, 1.03–5.22; *p* = 0.042) (Table 2). Upon multivariate Cox analysis, only the baseline hemoglobin level (HR, 0.73; 95% CI, 0.59–0.89; *p* = 0.002) and coronary artery involvement (HR, 4.11; 95%CI, 1.74–9.71; *p* = 0.001) retained associations with the incidence of MACCE. The detailed parameters of the univariate and multivariate Cox hazard analyses are shown in Table 2.

Univariate Cox proportional hazard analysis showed only a baseline white blood count as a prognostic factor in restenosis following EVT, as well as a trend toward significance when the patient was admitted in an emergency state with symptoms of cerebral ischemia or a dissecting artery (Table 3). Upon multivariate Cox analysis, only a baseline white blood count (HR, 1.24; 95%CI, 1.12–1.38; *p* < 0.001) retained associations with the incidence of restenosis. The detailed parameters of the univariate and multivariate Cox hazard analyses are shown in Table 3.

## 4. Discussion

### 4.1. Cerebral Ischemia as a Result of the Delay in Established Diagnosis

Approximately 5% to 15% of all strokes occur in adults aged 18 to 50 [42,43]. The causes or underlying pathogenesis of strokes in younger adults can be challenging to discern [42]. However, the incidence of stroke is increased in children and young adults. There is also growing evidence on the increased contribution of diabetes, hypertension, obesity, hyperlipidemia, and smoking among young people [44]. Strokes can complicate pregnancy, occurring in 30.0 per 100,000 pregnancies [45]. Among pregnancy-related strokes, aortic dissection Stanford type A and type B contribute significantly to stroke cases [46]. Among stroke patients between 18 to 50 years old, vascular diseases, like arterial dissection, moyamoya disease, cerebral venous thrombosis, reversible cerebral vasoconstriction syndrome, and TAK, have been more frequently found compared to non-young adults [43]. 

Between 10% to 25% of all ISs in patients below 50 years of age are associated with extracranial and intracranial arteriopathies [11,47,48,49]. However, the true incidence rates of TAK, FMD, and aortic arch variants are difficult to assess precisely. In a Taiwan study, the most common risk factors in children under the age of 18 were vascular diseases (26.3%), infection (14.0%), and cardiac disorders (9.1%) [50]. Among arteriopathies, moyamoya disease was one major cause of ischemic stroke (7.6%) [50]. TAK was reported in 3, CoA in 3, supraaortic stenosis in 3, and Ehlers–Danlos syndrome in 1 case out of a total of 685 stroke patients [50]. 

Carotid/vertebral dissection, TAK, and FMD account for 15.4%, 8.6%, and 1.9% of all cerebral ischemia cases in young adults between 19 and 45 years of age [51]. In TAK disease, stroke is common and might even be the presenting manifestation [52].

In FMD, TIA and IS seem to occur mainly in the presence of associated cervical artery dissection due to artery-to-artery thromboembolism or cerebral hypoperfusion [53]. The study by Atalay et al. showed that the prevalence of dissection among stroke hospitalizations was around 7% in young adults aged 18–40 [54]. Nedeltchev et al. reported cervical artery dissection as an underlying cause of stroke in 24% of their young stroke patients [55]. 

There are many different mechanisms behind aortopathies, like the bicuspid aortic valve, inflammatory diseases, FMD, and heritable connective tissue disorders (e.g., Ehlers–Danlos syndrome) [56,57]. It has been reported that bovine arch variation is associated with the occurrence of aneurysms or type B aortic dissection [57], as well as with an early age at stroke presentation (on average, 53 years old), compared to normal branching pattern (61 years) [18]. Furthermore, type B dissections, such as the one in our present study, often occur in patients with normal-sized or only mildly enlarged aortae, and, therefore, are unpredictable [46]. Malformative pathology of the aorta is often associated with congenital and/or valve diseases [56,57]. Aortic dissection and rupture are often consequences of disturbed shear stress. Some aortic arch variants and branching patterns, like bovine arch, CoA, vascular rings, aberrant subclavian artery, and bicarotid trunk, cause predisposition to stiffening of the aortic wall, thus promoting the growth of atherosclerosis and the formation of aneurysms [58]. Arterial stiffness is a key risk factor for cardiovascular mortality and morbidity [58,59]. 

Currently, when diagnostic work-ups are very well developed and reliable, some urgent admissions for cerebral and cardiovascular symptoms could be avoided. In the present study, 52 (63.4%) of 82 patients had presented with chronic symptoms for years, including chronic systemic inflammation, fevers, vision disturbances, impaired pulse over the radial artery, resistant hypertension, swelling discomfort and dysphagia, and claudication. These biochemical and clinical findings should alert physicians during routine ambulatory visits. Many of the signs and symptoms of TAK, FMD, and AAP are nonspecific, such as recurrent headaches, ocular disturbances, dysphagia, shortness of breath, and syncope [1,2,3]. This might require more diagnostic work-ups, but also should awaken awareness [4,8,13,57]. However, there continues to be an unacceptably long delay in the diagnosis of TAK, which is estimated at 2 to 3 years, and FMD, with an average delay of 4 to 9 years [1,2,60].

The delay in diagnosis results in late complications of TAK, FMD, and other NA-AAPs. In our retrospective study, 47.6% of the hospitalized patients were unscheduled emergency admissions, while in 64.5%, a recent cerebral ischemia had been documented. In line with this finding, the respective societies and registries have reported stroke and hemispheric transient ischemic attack as a presenting symptom among 15.6% of patients with FMD [12], 11.6% with TAK, and 22–25.7% with aortic arch and branching variants [1,2,12,61,62]. Consistently, retrospective clinical series and national registries for TAK, FMD, and other AAPs have reported between 40% to 70% of patients as having had one or more disease-related symptoms long before a diagnosis was established [1,2].

Mirouse et al. reported that a period from the development of the first symptoms to TAK diagnosis of >1 year (HR, 2.22 [1.30–3.80]; *p* = 0.005) was independently associated with cerebrovascular ischemic events, and that TAK patients with a history of TIA or stroke had a 4.5-fold higher risk of cerebrovascular ischemic event recurrence (95% CI, 2.45–8.17; *p* < 0.0001) [63]. The mechanism of cerebral ischemia in TAK includes blockage of the supraaortic artery due to an inflammatory process or thrombosis, carotid aneurysm/dissection, vascular steal, reduced cerebral blood flow, or embolization to the cerebral arteries [64]. In fact, patients with carotid artery stenosis frequently demonstrate insufficient cerebral collateral flow upon presentation with ischemic stroke [65,66]. 

In line with this, the most feared and serious sequelae of cerebrovascular FMD include TIA, stroke, subarachnoid hemorrhage, and cervical artery dissection. The frequency of neurological events in the US Registry for FMD was significant, including a hemispheric TIA in 13.4%, amaurosis fugax in 5.2%, stroke in 9.8%, and cervical artery dissection in 12.1% of patients [12]. The pathomechanism of cerebral events in FMD patients is complex, including severe stenosis producing cerebral hypoperfusion, embolization, thrombosis, dissection, and aneurysm rupture [12]. Recent data from the US Registry for Fibromuscular Dysplasia reported the prevalence of carotid artery dissection in patients with FMD to be around 16% [53].

Ischemic stroke or TIA can occur in patients with aortic dissection type A and type B. Neurological symptoms appear in 17% to 40% of patients with aortic dissection, especially those with type A aortic dissection, including the most frequent right hemispheric stroke (69.2–71%) [67,68]. There are two possible pathogenic mechanisms: (1) the dissection may block blood flow through the supraaortic trunks or advance towards them, or, less frequently, (2) a mural thrombus may cause artery-to-artery embolism if it reaches the true lumen [69,70]. In patients without trauma, frequent etiologies of aortic arch and side-branch dissection are variants in the branching pattern of the aortic arch, predisposing the patient to intimal tearing. Aortic arch dissection is more frequent in patients presenting with a bovine arch, CoA, Kommorell aneurysm, a bicarotid trunk, or arteria lusoria with aneurysm formation [71,72]. CTA and MRA results showed that, in about 10–25% of the examined individuals, the aortic arch’s branches did not follow the “normal/usual” branching pattern, with variations in their numbers and origins [22,73].

Ischemic stroke accompanying aortic dissection may reduce awareness of the stroke unit. The low level of consciousness and speech and language alterations may hinder or prevent the detection of acute chest pain [69]. In addition, chest X-ray showed mediastinal widening or aortic enlargement in only 50% of cases [74]. This explains the difficulty of detecting aortic dissections manifesting themselves in neurological symptoms and the consequent higher mortality rates (30%) compared to those seen in other cases of aortic dissection (22.6%) [69]. Missed diagnosis may also lead to thrombolysis, which is recommended now in ischemic stroke, with a mortality rate of 71% in patients receiving rTPA [75].

Recently, novel diagnostic tools have emerged that allow for the use of invasive angiography only when EVT is recommended. CTA and MRI techniques can replace standard angiography in class II, level of evidence A, according to the European League Against Rheumatism Committee of the American College of Rheumatology [29]. The comparison of CTA and MRA and catheter-based angiography for carotid stenosis has shown that the diagnostic accuracy is highly correlative, with an accuracy exceeding 97% for MRA and above 83% for CTA [74]. 

In addition, CDUS may offer important information regarding arterial wall thickening (a “macaroni” sign), lumen stenosis, flow pattern, and blood flow directions [23,66].

Eventually, non-invasive imaging modalities may guide EVT and surgical approaches when lesions cannot be resolved by a conservative approach.

### 4.2. Patients’ Management and EVT in Non-Atherosclerotic Aortic Arch Disease

The present study focused on the outcomes and long-term prognosis of EVT in patients with TAK, FMD, and other AAPs. As the etiology of these morbidities varies, the outcomes depend on the natural course of specific diseases. We have demonstrated that the EVT procedures were feasible, associated with an acceptable 30-day severe complication rate of less than 2.5%. Most patients underwent EVT with provisional stenting, which is particularly recommended in TAK patients (in our study, 86.7%). In the study conducted by Joseph et al., cerebral hyperperfusion was a significant problem in TAK patients referred to EVT when all arch branches were severely obstructed [28]. This is in line with our present and previous observations [76,77]. One of two major severe periprocedural complications was hyperperfusion syndrome in patients with TAK presenting with an occluded left common carotid artery and right subclavian artery, who were referred for recanalization of the occluded left subclavian artery.

In the FMD group, 50% of patients underwent stent implantation. This is in line with data from other endovascular centers. Similarly to the experiences of other centers, most FMD cases presented as acute cervical artery dissection. In our cohort of FMD patients, 14 (53.8%) patients had spontaneous cervical artery dissection upon hospital admission, and only in 3 patients was recanalization with subsequent stent-supported angioplasty feasible, provided that the true lumen of the carotid artery was identified correctly. The remaining patients were offered dual antiplatelet therapy, vitamin K antagonists, or thrombolytics intravenously. 

In the literature, there is much controversy on the adequate management of cervical artery dissection. Approximately, one-third of patients demonstrate complete healing of the carotid arteries (this occurred for 11.5% of patients in our cohort) [78]. The benefit of emergent stenting in addition to thrombectomy in tandem occlusions for cases of internal carotid artery dissection remains unclear [78,79,80]. Antiplatelet or anticoagulation therapy is also used, although more studies are needed to reach a consensus on secondary stroke prevention [79,80]. In the Cervical Artery Dissection in Stroke Study (CADISS) and the Aspirin versus Anticoagulation in Cervical Artery Dissection study (TREAT-CAD), which compared antiplatelet therapy with anticoagulation in the acute treatment of patients with extracranial cervical artery dissection, there was no difference in the rate of recurrent stroke in the two groups [81].

In the Swiss Registry, among 62 cervical artery dissection patients (median age 48.8 years), 24 received intravenous thrombolysis and 38 received EVT (with or without prior intravenous thrombolysis) [82]. The EVT included either mechanical thrombectomy, thrombus aspiration, or intra-arterial thrombolysis with or without stent placement. Recanalization was achieved in 84.2% EVT patients and 66.7% intravenous thrombolysis patients (*p* = 0.278), while periprocedural death was reported in 6 (15.8%) EVT patients (*p* = 0.045) [82]. However, a meta-analysis of the six studies reporting on death at three months, which was stratified to the treatment groups, did not show a significant difference between intravenous thrombolysis and EVT patients (OR 0.81, 95% CI [0.16–3.97] [82]). 

### 4.3. Restenosis

We have observed that the main drawback of EVT procedures is RS, which is particularly high in TAK patients, but it was also encountered in FMD patients. Restenosis-free survival rates were 78.3%, 52.2% and 45.4% for the TAK group and 100%, 84.3%, and 75.8% for the FMD group at 1, 3, and 5 years (log rank *p* = 0.029), respectively. We did not observe restenosis in the other AAPs group, although new aneurysms and aortic dissection propagation were observed in three patients with aortopathies. In our present study, we observed that a baseline white blood count was the only independent risk factor for restenosis (HR; 1.25, 95% CI; 1.12–1.39, *p* < 0.001). 

These results are in line with data from other centers [28,83,84]. High restenosis rates following vascular intervention ranged from 37% to 62%, usually obviating prophylactic intervention in TAK [78]. However, in a Korean study, the restenosis rate was lower, i.e., 10% of TAK cases after a mean follow-up of 34 months [85]. On the other hand, Sharma and Gupta observed a 5-year patency rate of 67% in 264 TAK patients who had undergone EVT for the renal artery [84]. In a large single-center registry of 1149 patients treated for TAK during a 26-year period (1996–2022), Joseph et al. performed EVT procedures for the subclavian or axillary artery in 630, the carotid artery in 333, and the vertebral arteries in 39 patients [28]. In this study, the restenosis-free patient rate was 48.6% after primary EVT, with occlusive restenosis as a large contributor. The patency rate increased to 83.3% after repeated EVT procedures for restenosis [28]. The recurrent restenosis in TAK patients outranged that seen in patients with atherosclerotic lesions [86,87]. On the contrary, there is a lack of clinical data on the incidence of restenosis following EVT in carotid FMD [88]. At the same time, the recurrence rate of cervical artery dissection is rather low. The Kadian-Dodov et al. study showed that out of 52 dissections, only 3 patients (5.8%) experienced a new dissection and none of the patients developed a new aneurysm after a mean follow-up period of 35.3 months [89].

Clinical data indicate that active inflammatory status is a main driving factor for restenosis [90,91,92]. This finding is in agreement with the conclusion drawn by Saadoun et al. that active inflammation at the time of revascularization, defined as erythrocyte sedimentation rate (ESR) > 30 mm/h and CRP > 6 mg/L, increases the likelihood of cardiovascular complications during the follow-up period [93]. However, accumulating evidence indicates that ESR and CRP show moderate performances in terms of distinguishing active TAK [92]. Numerous novel scores and biomarkers, like the Disease Extent Index in TAK (DEI.TAK) and the Indian TAK Clinical Activity Score (ITAS2010), PET-CT, MRA, and circulating biomarkers, suggest a better assessment of active disease [92]. 

Consistently, the main clinical issue in patients with TAK is obtaining the remission of inflammatory processes, which is associated with therapy failure in a substantial number of patients [90,91,92]. Steroids are the mainstay in immunosuppressive treatment [91]; however, disease relapses and steroid dependence are frequent in TAK [94]. In such cases, pharmacological treatment requires the use of adjunctive or alternative drugs like azathioprine; methotrexate; cyclophosphamide; mycophenolate mofetil; or monoclonal antibodies, i.e., antagonists of TNF-alfa (infliximab), soluble IL-6 receptor (tocilizumab), or CD20 (rituximab). The preferred combination of drugs largely depends on the individual center’s experience [91,92]. 

### 4.4. Major Adverse Cardiac and Cerebral Events in Patients with AAPs

Our study has demonstrated that non-atherosclerotic pathology of the aortic branch and/or its major side-branches constitutes a rare, but important, cause of vascular and cerebral complications. At our institution, patients with AAPs constituted 1.07% of all patients referred for EVT. We observed one periprocedural death during the hospital stay, accounting for 1.2% of cases. 

However, in the median 64 months of follow-up, MACCE occurred in 24 (29.6%) patients, including 5 CVD, 13 non-fatal ISs, and 6 non-fatal MIs. We noticed the highest rate of MACCE in TAK patients (39.5%), followed by the AAPs group (29.4%), with the lowest rate in the FMD group (15.4%) (*p* = 0.078). Upon multivariate Cox analysis, a baseline hemoglobin level (HR, 0.73; 95%CI, 0.59–0.89; *p* = 0.002) and coronary artery involvement (HR, 4.11; 95%CI, 1.74–9.71; *p* = 0.001) showed associations with the incidence of MACCE.

Park et al. showed that TAK was associated with a significantly increased risk of mortality, with cardiovascular disease as the most common cause of death, accounting for 29 of the 64 deaths (45.3%) due to heart disease and stroke, followed by neoplasms in 9 patients (14.1%) [95]. Clinical studies have reported the high mortality rate of TAK, estimated to be up to 35%. We observed a significantly lower incidence of cardiovascular death in patients with TAK, accounting for 7.8%, although coronary involvements were found to be an independent risk factor for MACCE. Our results are closer to those of the study of Mirouse et al., who reported incidence of death in 16 (5%) out of 318 TAK patients in a median [IQR] follow-up of 6.1 [2.8–13.0] years [96]. In the aforementioned study, the main causes of death included mesenteric ischemia (25%) and aortic aneurysm rupture (25%) [96]. Interestingly, Misra et al. reported a similar risk of mortality in TAK patients with and without stroke/TIA (HR; 1.38, 95% CI 0.19–10.20) after adjustment for gender, age of disease onset, delay to diagnosis, baseline disease activity, and number of conventional or biologic/targeted synthetic immunosuppressants used [97]. Thus, stroke and TIA do not appear to adversely affect survival in TAK patients [97]. On the contrary, the presence of a metabolic syndrome was detected to be an independent risk factor for CVD (*p* < 0.001) [98]. Ideally, endovascular interventions should be performed during periods of remission, as the complication and mortality rates are higher when the disease is active [93,99,100,101]. 

The next group of disorders associated with nearly 30% incidence of MACCE in our study were arteriopathies. Aortic arch pathologies are uncommon and often pose significant challenges to surgical or endovascular treatment. The Ontario Stroke Registry followed young stroke patients for approximately 5 years and found that they had a hazard ratio of 5.2 at 5 years for the occurrence of one of these events, whereas among older stroke patients, the 5-year hazard ratio was only 1.3 compared with their matched controls [102]. However, this cohort of patients lacks the systemized methods used for prognostic studies on mortality among patients with aortic dissection, due to the group’s small size and the high risk of bias [103].

As is rare but life-threatening, sudden onset and symptom presentation with cerebral ischemia or aortic arch dissection is observed in Ehlers–Danlos syndrome type IV, associated with abnormal type III collagen [20]. This type of the Ehlers–Danlos syndrome is characterized by sudden, unexpected arterial rupture (often in previously normal-sized arteries), easy bruising, hypermobility of small joints, and varicose veins. This scenario was observed in a 26-year-old primigravid female patient, who was admitted on the 10th day after uneventful delivery of a child with acute chest pain, cerebral ischemia symptoms, and cardiogenic shock. The emergency CTA revealed dissection of the aortic arch arising from the left common carotid artery and ending at the level of the renal arteries. It is likely that an emergency CTA followed by an immediate stent-graft implantation saved her life. The patient with Ehlers–Danlos syndrome represents one of four young female patients who underwent emergency EVT procedures during the peri-partum and post-partum periods in our study. New aneurysms developed in two out of four patients, which indicates the need for watchful follow-up in these groups of patients. 

Advanced diagnostic work-ups with CTA and MRI enables quick identification of progressive lesions, which cause new ischemic symptoms. They are also mandatory in order to diagnose and monitor other vascular diseases, including arteriovenous fistulas, vasculitis like Kawasaki disease, and aneurysmal disease. In particular, Vein of Galen malformations, i.e., congenital arteriovenous fistulas, can present as heart failure and hydrocephalus in neonates [104]. The recognition and management of this malformation is challenging. CTA can provide a more detailed view of vasculature compared to MRI. Therefore, it may facilitate and guide embolization during EVT, which usually takes place around 6 months of age. If this is not effective, gamma knife radiotherapy may reduce malformation size and many feeders, optimizing the outcomes [104].

### 4.5. Novel Perspectives in NA-AAPs Management

#### 4.5.1. Artificial Intelligence

Novel technologies, like artificial intelligence (AI) and related tools like machine learning, have significant potential to impact the accuracy and speed of diagnosis, as well as to improve the prediction of the outcomes of cardiovascular diseases [105,106]. Numerous studies have already highlighted the advantages of AI-based approaches in the diagnostic imaging of carotid artery diseases [107] and aortic pathologies [108]. Furthermore, Hayıroğlu et al. demonstrated that AI algorithms can enhance healthcare professionals’ ability to diagnose coronary artery disease by analyzing imaging data from CTA and MRI. According to the study, a deep learning system can precisely identify and evaluate coronary artery stenosis on CT images [109]. Manual measurement of the aortic diameter is time-consuming and may be inaccurate due to inter-reader variability, depending on the specific plane and location where the reader is measuring [110]. Recently, a very elegant study by Artzner et al. demonstrated high accuracy of AI-based machine learning in the diagnosis of thoracic aortic aneurysms [110]. In patients with Stanford type A and/or B dissections, the segmentation tool measured the true and false lumen together if both had blood flow [110]. In this study, the inter-reader agreement between the algorithm and the mean of the radiologists was excellent for each dedicated location, with an ICC between 0.961 (95% CI: 0.940–0.974) in the mid-ascending aorta and 0.984 (95% CI: 0.977–0.990) in the mid-descending aorta [110]. However, further studies are warranted in order to fully reveal the potential application of AI in the diagnosis and prognosis of TAK, FMD, and other AAPs.

#### 4.5.2. Molecular and Cellular Biomarkers

The clinical and molecular presentation of TAK is highly variable. The gold standard for the diagnosis and accurate assessment of disease activity (involving imaging and laboratory tests showing adequate sensitivity or specificity) is still lacking. Thus, the potential role of molecular and cellular biomarkers in TAK diagnosis and management has been intensively investigated recently.

Acute phase response molecules, i.e., erythrocyte sedimentation rate (ESR) and C-reactive proteins (CRP), have been used to monitor TAK activity in clinical settings, although they have failed to reliably quantify disease activity [111]. Inflammation in TAK may also be reflected by elevated white blood cell counts, complement (C3, C4, CH50) levels, fibrinogen, and immunoglobulin G [112]. Recent data, however, have shown that elevated ESR, CRP, and IL-6 are associated with active disease, lower possibility, and longer time to achieve disease remission [113]. Elevation of any among ESR, CRP, IL-6, and TNFα is associated with high risk and a short time to relapse during follow-up [114]. 

Recently, several promising biomarkers have been investigated in terms of distinguishing active vs. inactive TAK [114]. One of those, pentraxin-3 (PTX3), has been shown to be more sensitive and less specific, with overall better performance than CRP (AUC 0.914 vs. 0.905), in distinguishing active TAK. Moreover, the levels of pentraxin-3 did not correlate with the dose of prednisolone [114]. Cui X et al.’s group built a logistic regression model based on multiple cytokines, including CA (cancer antigen) 125, FLRG (follistatin-related protein), IGFBP (insulin-like growth factor-binding protein)-2, CA15-3, GROa (growth-regulated alpha protein), LYVE (lymphatic vessel endothelial hyaluronic acid receptor)-1, ULBP (UL16-binding protein)-2, and CD 99, with an area under the curve reaching 0.909 for discriminating TAK activity status [115]. Dong H and colleagues showed that the expression level of C-C chemokine ligand CCL2, CCL20, CXCL8, and CXCL10 was elevated in active TAK patients, indicating that these chemokines might act as potential biomarkers in evaluating the disease activity [116].

The review by Savioli B et al. indicates that interleukin IL-6 is the cytokine that best reflects disease status and disease activity in TAK [117]. The authors pointed out that all studies that evaluated IL-6 serum/plasma levels showed significantly higher levels in TA patients compared with healthy controls, and, in the majority of them, active disease was associated with higher IL-6 levels than the remission state. Thus, IL-6 is the central cytokine involved in the pathophysiology of TAK [117].

In the search for new autoantibodies that could be adopted in a clinical setting for early TAK diagnosis, Wen X et al.’s group identified and validated eight novel autoantibodies as TAK-specific biomarker candidates, three of which (SPATA7, QDPR, and PRH2) were confirmed to be TAK-specific autoantigens [118].

The infiltration of macrophages was one of the key findings in the histopathology of TAK [119]. Kong X et al. showed that macrophages contribute to vascular pathological changes in TAK by undergoing phenotype transformation [119]. Moreover, CCL2 was found to be an important factor for recruiting macrophages and a potential biomarker of disease activity. In the study by de Aguiar MR et al., the investigators evaluated monocyte subsets and monocyte-related chemokines in the peripheral blood of TAK patients and healthy controls [120]. They showed that TAK was associated with altered counts of monocyte subsets in the peripheral blood compared to healthy controls, and that CCL22 was the chemokine with the strongest association with active disease in TAK.

TAK is not solely a unique disease characterized by inflammation of the major arterial branches that causes narrowing of the arteries or aneurysms. It is important to mention acute arteritis, which occurs in children around 5 years of age [121]. In Kawasaki disease, similarly to TAK, the inflammatory process is complex, and IL-6, IL-8, TNF-α, IFN-γ, and CRP are involved in the course of the disease. Data suggests that dysregulation of the inflammatory response is involved in the disease progression and enlargement of aneurysms’ [121]. Particularly, high levels of neutrophils facilitate the infiltration of arterial vessels, resulting in necrosis and aneurysms. As large aneurysms tend to persist for years, events of sudden aneurysm rupture are not uncommon. Therefore, the prognostic markers of adverse prognosis are important to identify. A systematic review and meta-analysis of seventeen studies indicated an important role of the elevated levels of the neutrophil-to-lymphocyte ratio on the formation of coronary abnormalities and aneurysms in patients with Kawasaki disease [121]. In line with this, a recently published study showed the specific patterns of the endothelial cell response that leads to the destruction of vascular smooth muscle cells and arterial structures, progressing, in consequence, to the specific cardiovascular outcomes of Kawasaki disease [122].

This study had several limitations. First, this was a single-center, retrospective study. Due to the diseases’ rarity, the number of patients was limited. Therefore, larger-scale, preferably multi-center, studies would be useful.

In conclusion, this study shows that AAPs should not be neglected in the clinical setting, as they can be life-threatening conditions requiring multidisciplinary approaches. The knowledge on prognostic risk factors for adverse outcomes may improve surveillance in this group of patients. Follow-up of subjects with TAK undergoing EVT appears to be critical, as the restenosis rate is high. Cardiovascular events are numerous, particularly in the TAK and aortopathy groups. The outcome is mainly driven by the activity of inflammatory processes.

## Figures and Tables

**Table 1:** Patients’ characteristics, diagnostic work-ups, and procedures.

Aortic Arch Pathology	Takayasu Arteritis N = 38	Fibromuscular Dysplasia N = 26	Other AAP N = 18	*p*-Level (ANOVA)
Demographic data				
Age, mean (SD)	43.2 (13.5)	50.1 (15.3)	46.4 (25.7)	0.187
Female gender, n (%)	35 (91.9)	17 (65.3)	13 (72.2)	0.024
In post- or peri-partum period, n (%)	0 (0)	1 (3.8)	4 (22.2)	0.028
Emergency hospital admission, n (%)	11 (28.9)	16 (61.5)	12 (66.6)	0.006
Recent ischemic stroke, n (%)	23 (60.5)	20 (76.9)	13 (72.2)	0.362
Cervical artery dissection, n (%)	1 (2.6%)	14 (53.8)	3 (16.7)	<0.001
Body mass index, kg/m^2^ (SD)	24.0 (3.1)	24.5 (2.8)	25.1 (1.38)	0.396
Hypertension, n (%)	28 (73.6)	20 (76.9)	13 (72.2)	0.931
Hyperlipidemia, n (%)	23 (60.5)	12 (46.1)	9 (50)	0.504
Diabetes mellitus type 2, n (%)	11 (28.9)	3 (11.5)	3 (16.6)	0.220
Past or present smoking, n (%)	14 (36.8)	2 (7.6)	4 (22.2)	0.026
Chronic kidney disease *, n (%)	9 (23.6)	7 (26.9)	5 (27.7)	0.139
Systemic inflammation, n (%)	38 (100)	1 (3.8)	0 (0)	n/a
Other immune-mediated disease, n (%)	11 (28.9)	1 (3.8)	0 (0)	0.012
Ehlers–Danlos syndrome	0 (0)	0 (0)	1 (5.5)	0.789
Cardiac valve involvement, n (%)	9 (3.6)	0 (0)	1 (5.5)	0.011
Pericardial effusion, n (%)	4 (10.5)	0 (0)	0 (0)	0.333
Myocarditis, n (%)	5 (13.1)	0 (0)	0 (0)	0.189
Coronary involvement, n (%)	15 (39.4)	3 (11.5)	3 (16.6)	0.025
Pulmonary involvement, n (%)	1 (2.6)	0 (0)	1 (5.5)	0.789
Abdominal involvements ^#^, n (%)	30 (78.9)	16 (61.5)	6 (33.3)	0.004
Coarctation of the aorta, n (%)	4 (10.5)	0 (0)	3 (16.6)	0.343
Hypoplasia of the aortic arch, n (%)	0 (0)	0 (0)	1 (5.5)	0.789
Intracranial aneurysm, n (%)	2 (5.2)	2 (7.6)	1 (5.5)	0.918
Biochemical data				
C-Reactive protein, (mg/L), mean (SD)	14.38 (15.6)	1.41 (1.12)	25.8 (36.3)	0.003
White blood count, mean (SD)	10.1 (3.62)	6.86 (1.91)	7.38 (2.45)	<0.001
Hemoglobin (g/L), mean (SD)	12.6 (2.1)	13.7 (1.33)	12.2 (1.87)	0.029
Serum creatinine (µmol/L), mean (SD)	78.3 (22.8)	79.8 (23.7)	78.9 (29.6)	0.962
D-dimers (g/L), mean (SD)	387 (56)	102 (11.1)	2159 (1642)	0.010
Triglycerides (mmol/L), mean (SD)	1.53 (0.6)	0.97 (0.37)	1.46 (0.62)	0.001
LDL-C (mmol/L), mean (SD)	3.04 (1.1)	2.22 (0.73)	3.13 (0.90)	0.007
HDL-C (mmol/L), mean (SD)	1.47 (0.51)	1.52 (0.33)	1.29 (0.32)	0.303
Diagnostic imaging work-ups				
Brain CT/MRI, n (%)	38 (100)	26 (100)	18 (100)	n/a
CDUS, n (%)	38 (100)	26 (100)	18 (100)	n/a
Angio-CT, n (%)	36 (94.7)	23 (88.5)	17 (94.4)	n/a
Angio-MRI, n (%)	2 (5.2)	3 (11.5)	1 (5.5)	n/a
Angiography, n (%)	38 (100)	26 (100)	18 (100)	n/a
Applied treatment for the aortic arch and branches				
Endovascular treatment, n (%)	33 (86.8)	13 (50)	13 (72.2)	0.006
Carotid artery, n (%)	20 (52.6)	15 (57.7)	2 (11.1)	0.004
Innominate or subclavian artery, n (%)	21 (50)	0 (0)	5 (27.8)	<0.001
Procedure for more than one vessel, n (%)	8 (21.1)	2 (7.6)	1 (5.5)	0.165
Balloon angioplasty alone, n (%)	0 (0)	1 (3.8)	0 (0)	n/a
Stent implantation (per procedures), n (%)	41	14	7	n/a
Stent graft, n (%)	0 (0)	0 (0)	7 (38.9)	n/a
Periprocedural CVD	0 (0)	0 (0)	1 (5.5)	0.791
Periprocedural HPS	1 (2.6)	0 (0)	0 (0)	0.463
No revascularization, n (%)	5 (13.2)	13 (50)	5 (27.8)	0.003
Steroids, n (%)	38 (100)	1 (3.8)	0 (0)	<0.001
Other anti-inflammatory drugs, n (%)	17 (44.7)	0 (0)	0 (0)	<0.001
Biological treatment (tocilizumab), n (%)	4 (10.5)	0 (0)	0 (0)	0.333
Antiplatelet(s) therapy, n (%)	38 (100)	24 (92.3)	14 (77.8)	0.012
Vitamin K antagonists, n(%)	3 (7.8)	2 (7.6)	2 (11.1)	0.906
New oral anticoagulants, n (%)	0 (0)	0 (0)	0 (0)	n/a
Actylise, n (%)	0 (0)	3 (23.1)	0 (0)	n/a
Outcomes, N *	38	26	17	
Follow-up, mean, months (SD)	76 (37)	79 (45)	53 (35)	0.043
Restenosis, n (%) **	19/33 (57.6)	2/13 (15.4)	0 (0)	0.019
Re-PTA, n (%)	16/17 (94.1)	2 (7.6)	0 (0)	0.417
MACCE, n (%)	15 (39.5)	4 (15.4)	5 (29.4)	0.113
CVD, n (%)	3 (7.8)	0 (0)	2 (11.7)	0.260
Non-fatal MI, n (%)	3 (7.8)	2 (7.6)	1 (5.8)	0.948
Non-fatal IS, n (%)	9 (23.6)	2 (7.6)	2 (11.7)	0.187

* One patient was not included due to periprocedural death. ** Numbers were calculated per endovascular procedure. ^#^ Abdominal involvement includes celiac trunk, mesenteric and renal arteries involvements. AAP—aortic arch pathology; CT—computed tomography; CVD—cardiovascular death; IS—ischemic stroke; MACCE—major cardiac and cerebral event; MI—myocardial infarction; MRI—magnetic resonance imaging.

**Table 2:** Univariate and multivariate Cox proportional hazard analysis for the incidence of major cardiac and cerebral events following endovascular treatment.

	Univariate Cox Proportional Hazard Analysis			Multivariate Cox Proportional Hazard Analysis		
Clinical Parameter	Hazard Ratio	95% Confidence Interval	*p*-Value	Hazard Ratio	95% Confidence Interval	*p*-Value
Age	1.03	1.01–1.07	0.019	1.03	0.99–1.06	0.080
Female gender	1.22	0.45–3.32	0.696			
Emergency admission	0.61	0.26–1.44	0.261			
Recent cerebral ischemia	1.09	0.44–2.70	0.845			
Etiology	1.11	0.39–3.12	0.182			
Hypertension	1.12	0.38–3.36	0.833			
Diabetes mellitus type 2	1.37	0.55–3.41	0.493			
Hyperlipidemia	1.29	0.54–3.07	0.561			
Smoking	1.94	0.84–4.50	0.122			
Body mass index	1.06	0.92–1.21	0.426			
Coronary artery involvement	2.31	1.03–5.22	0.042	4.11	1.74–9.71	0.001
Peripheral arterial disease	1.92	0.85–4.36	0.114			
Renal artery involvement	1.12	0.48–2.62	0.790			
Baseline creatinine	1.00	0.99–1.02	0.620			
Baseline Hemoglobin level	0.78	0.64–0.96	0.019	0.73	0.59–0.89	0.002
Baseline hs-CRP	1.00	0.99–1.02	0.610			
Baseline white blood count	1.10	1.01–1.21	0.045	1.01	0.91–1.11	0.955
Baseline LDL-cholesterol	1.21	0.86–1.72	0.273			
Baseline HDL-cholesterol	0.47	0.15–1.42	0.179			
Tryglicerydes	1.11	0.58–2.12	0.758			
In-stent restenosis	1.42	0.58–3.51	0.444			

**Table 3:** Univariate and multivariate Cox proportional hazard analysis for the incidence of restenosis following endovascular treatment.

	Univariate Cox Proportional Hazard Analysis			Multivariate Cox Proportional Hazard Analysis		
Clinical Parameter	Hazard Ratio	95% Confidence Interval	*p*-Value	Hazard Ratio	95% Confidence Interval	*p*-Value
Age	1.01	0.99–1.04	0.356			
Female gender	0.84	0.24–2.99	0.798			
Emergency admission	2.39	0.88–6.47	0.086	2.39	0.87–6.58	0.091
Recent cerebral ischemia	1.15	0.46–2.89	0.756			
Etiology	3.94	0.21–76.8	0.365			
Hypertension	1.35	0.46–4.01	0.583			
Diabetes mellitus type 2	0.73	0.24–2.15	0.564			
Hyperlipidemia	1.35	0.55–3.31	0.514			
Smoking	0.96	0.40–2.29	0.929			
Body mass index	0.98	0.81–1.18	0.836			
Coronary artery involvement	1.11	0.38–3.15	0.857			
Peripheral arterial disease	1.19	0.50–2.84	0.694			
Renal artery involvement	0.61	0.25–1.45	0.262			
Baseline creatinine	0.99	0.97–1.01	0.330			
Baseline hemoglobin level	1.03	0.82–1.29	0.819			
Baseline hs-CRP level	1.02	0.98–1.05	0.232			
Baseline white blood count	1.24	1.12–1.38	<0.001	1.25	1.12–1.39	<0.001
Baseline LDL-cholesterol	1.11	0.79–1.57	0.548			
Baseline HDL-cholesterol	1.51	0.53–4.34	0.440			
Tryglicerydes	0.90	0.42–1.90	0.782			

## Data Availability

Not applicable.
